# Interdisciplinary perspective-based behavioral prediction of e-cigarette use: A population-based study among Chinese college students

**DOI:** 10.18332/tid/204743

**Published:** 2025-07-04

**Authors:** Yu Chen, Zining Wang, Shaoying Jiang, Yujiang Cai, Jing Xu, Ying Wang

**Affiliations:** 1 School of Art and Communication, Fujian Polytechnic Normal University, Fuqing, China; 2 School of Journalism and Communication, Peking University, Beijing, China; 3 School of Culture and Law, Fujian Polytechnic Normal University, Fuqing, China; 4 School of International Studies, Peking University, Beijing, China; 5 School of Humanities and Communication, Zhejiang University of Finance and Economics, Hangzhou, China

**Keywords:** college students, e-cigarette use, media use, susceptibility, behavioral prediction

## Abstract

**INTRODUCTION:**

E-cigarette use is rising among young adults globally, and college students are particularly vulnerable due to high social media engagement and targeted promotions. Understanding which factors predispose this population to initiate vaping is critical for designing effective prevention strategies.

**METHODS:**

We conducted a cross-sectional survey of 303 never-smoking, never-vaping Chinese college students (aged 18–24 years) recruited via online platforms and referrals. The 25-item questionnaire assessed six domains: demographics, parental smoking, peer e-cigarette use, ‘quasi-deviant’ behaviors (regular alcohol consumption and bar attendance), social media use and trust, and exposure to e-cigarette marketing across five media channels. A three-item susceptibility scale was combined into a single index via principal component analysis. An Extremely Randomized Trees classifier (n_estimators=60, max_depth=6) with grid-search and five-fold cross-validation on a 75:25 train-test split, identified the strongest predictors of high susceptibility. Model performance was evaluated by accuracy and area under the receiver operating characteristic curve (AUC).

**RESULTS:**

The model achieved 81% classification accuracy. Feature importance (FI) indicated that bar attendance (FI=0.21), alcohol consumption frequency (FI=0.12), exposure to e-cigarette marketing messages (FI=0.08), social media use (FI=0.08), peer e-cigarette use (FI=0.05), and parental smoking (FI=0.05) were the most influential predictors. Among the participants, 18.8% were classified as high-susceptibility, indicating elevated risk for future vaping initiation.

**CONCLUSIONS:**

‘Quasi-deviant’ behaviors (regular alcohol use and bar attendance), social media marketing exposure, and social influences (peer and parental smoking) are key predictors of e-cigarette susceptibility in Chinese college students. Multi-level prevention strategies – enforcing digital marketing restrictions, peer-focused education, and integrated substance-use interventions – may effectively reduce susceptibility and avert vaping initiation in this high-risk group.

## INTRODUCTION

The use of electronic cigarettes has increased rapidly over the years^1^, research has shown that they still contain nicotine and produce toxic chemicals that pose health risks^2^. The aerosol from heating propylene glycol and vegetable glycerin can generate harmful substances such as formaldehyde and acrolein, which irritate the respiratory system^3^. Youth and young adults – whose brains and lungs are still developing – are particularly vulnerable to nicotine addiction and adverse effects^4^. Indeed, nicotine exposure during adolescence can impair brain development, and e-cigarette use by never smokers doubles the likelihood of subsequent smoking initiation^1,2^.

Globally, the use of e-cigarettes among young people has surged over the past decade^5^. In the United States, for example, the prevalence of past-30-day e-cigarette use among high school students climbed to an all-time high of 27.5% in 2019 before modest declines in recent years^5^. One driver of this epidemic is aggressive marketing and advertising by e-cigarette companies^4,6^. E-cigarette advertising employs themes and tactics similar to those long used to promote cigarettes (e.g. glamour, independence, social appeal) and leverages media channels popular with youth^6^. According to the US CDC, 7 in 10 middle and high school students reported exposure to e-cigarette marketing in 2021, with about 74% of social media users seeing vaping-related posts^6^. This pervasive marketing – much of it on social media and other digital platforms – has been strongly linked to youth vaping initiation. College students, who are typically active on social media and form a key target demographic for e-cigarette companies, are at high risk of such exposure.

In China, the regulatory environment for e-cigarettes has rapidly evolved^7^. Since 1 October 2022, China has banned the sale of flavored e-cigarettes, allowing only tobacco-flavored products in an effort to curb youth appeal^8^. Despite these regulations, an illicit market of flavored e-cigarette products disguised as everyday items (e.g. ‘milk tea cup’ or ‘cola can’ designs) persists on social media and e-commerce platforms^8^. This means Chinese college students can still be exposed to enticing e-cigarette content online, potentially increasing their curiosity and susceptibility. National surveys indicate that e-cigarette use among Chinese youth remains relatively low compared to Western countries (for instance, 6.5% of Chinese young adults aged 18–24 years were current e-cigarette users in 2023)^9^, but awareness is high and use is on the rise^10^. Given this window of opportunity for prevention, it is crucial to identify which factors most strongly predispose Chinese college students to initiating e-cigarette use.

Prior studies across various disciplines have highlighted a range of risk factors for youth vaping. From a health communication perspective, exposure to e-cigarette marketing and favorable information on media (both traditional and social media) is known to increase e-cigarette susceptibility^11^. From a social and behavioral perspective, influences from important others – such as family and friends – play a significant role: having close friends who use e-cigarettes or cigarettes has been associated with a substantially higher intention to vape^12,13^. Sociology research on deviance suggests that engagement in certain ‘quasi-deviant’ behaviors (those deviating from typical norms but not necessarily criminal) like underage drinking or frequenting bars/clubs often clusters with tobacco use in young people^14,15^. In college populations, studies have found that students who binge drink or party frequently are more likely to experiment with e-cigarettes. On the other hand, psychology and public health research has established ‘susceptibility to smoking’ – defined by Pierce et al.^16^ as the absence of a firm decision not to smoke – as a robust predictor of future tobacco initiation among never smokers. This susceptibility concept, initially developed for cigarettes, has been extended to e-cigarettes: youth who are deemed susceptible to vaping (e.g. those who answer ‘maybe’ or ‘unsure’ about future e-cigarette use) are significantly more likely to start vaping later^3,17-19^.

However, much of the existing literature examines these factors in isolation within a single discipline. Few studies have attempted to integrate multiple perspectives to predict e-cigarette use behavior in a holistic way. We posit that an interdisciplinary approach – combining influences from media exposure, social environment, personal behaviors, and psychological propensity – can improve the prediction of e-cigarette uptake among never-smoking youth. In particular, using susceptibility as an outcome measure provides a way to identify high-risk individuals before they initiate use^18^. Susceptibility is typically measured with only 2–3 survey items, making it a convenient screening tool, and it has demonstrated predictive validity for smoking initiation in diverse populations^3,16^.

Building on these insights, the present study aimed to identify key behavioral and environmental predictors of e-cigarette use susceptibility among Chinese college students, from an interdisciplinary perspective. We combined factors from communication (media and marketing exposure), sociology (peer and parental influences; ‘quasi-deviant’ behaviors), and public health (tobacco-related attitudes like susceptibility) in a single predictive model. Using a machine learning classification approach, we sought to determine which factors (or combination of factors) best distinguish high-susceptibility individuals. Ultimately, our goal is to inform targeted interventions by pinpointing modifiable risk factors that, if addressed, could help prevent e-cigarette initiation in this vulnerable population.

## METHODS

### Study design and participants

This research was a cross-sectional, survey-based study conducted from February to April 2023 among college students in China. The target population were current college/university students, and focused on never smokers and never vapers: inclusion criteria required that participants had never smoked combustible cigarettes and never used e-cigarettes at the time of the survey. Individuals who had experimented with smoking only once or a few puffs in the past (sometimes termed ‘one-puff smokers’) were excluded from the final sample, to ensure our cohort represented truly non-smoking, non-vaping youth. This exclusion was made because even minimal smoking experimentation can indicate lower resistance to tobacco, and such individuals have technically initiated tobacco use (albeit briefly), which could confound the measurement of susceptibility among never users. We initially recruited 350 eligible students. After excluding cases with inattentive or patterned responses on the questionnaire (e.g. unrealistically consistent answering patterns) and those meeting the ‘one-puff’ exclusion, a total of 303 valid participants were included in the analysis.

### Recruitment and data collection

We utilized a convenience sampling strategy with efforts to ensure geographical diversity. Participants were recruited through online advertisements on social media platforms popular with Chinese students (e.g. WeChat, Weibo) and via word-of-mouth referrals. Interested individuals contacted the research team and were screened for eligibility. Qualified participants provided informed consent prior to participation; for those under the age of 18 years, parental consent was also obtained. Data were collected through one-on-one online interviews conducted by trained researchers using a video conferencing platform. During each interview, the researcher presented the survey questions to the student and recorded the responses in real time. This interviewer-administered approach was chosen to clarify any questions and avoid misunderstandings in interpreting survey items. Each interview lasted approximately 20–30 minutes. Participants were assured of their anonymity and that there were no right or wrong answers, in order to reduce social desirability bias in responses. Upon completion, participants received a small incentive (equivalent to US$5) for their time.

### Measures

The survey instrument was developed by the research team drawing from several established sources, including the Global Adult Tobacco Survey (GATS) and Global Youth Tobacco Survey (GYTS) questionnaires for core tobacco use items, as well as prior studies on e-cigarette risk factors.

As a strong predictor, susceptibility was treated as the dependent variable in this study to reflect the tendency of the youth to use e-cigarettes. Based on the commonly used 2-tiem measurement, this study made improvements to realize Chinese context-based measurement. Specifically, susceptibility was measured on a 4-point Likert scale including the following three questions: ‘Have you tried using e-cigarettes recently?’, ‘Do you think you will try using e-cigarettes in the next year?’, and ‘If your best friend offered you an e-cigarette in the next year, would you use it?’. Therefore, in the extraction of the susceptibility variable, instead of the traditional method of taking the average value, this study used principal component analysis (PCA) to achieve data dimension reduction and make the new variable better explain the variances in the three questions.

In addition to basic demographic information, the questionnaire included dimensions such as ‘quasi-deviant’ behaviors, smoking-related behaviors of significant others^20^, exposure to e-cigarette marketing information, frequency of media use, trust in different media, and e-cigarette susceptibility. In the questionnaire, the smoking habits of family and friends and the frequency of visiting bars are captured through ‘yes or no’ single-choice questions. The frequency of consumption is measured using a scale of five options: never, ≤ once a month, 2–4 times a month, 2–3 times a week, and ≥4 times a week.

For participants’ exposure to e-cigarette marketing information in various media environments, this study divided it into five categories: print media, radio and television media, websites, domestic social media, and foreign social media. A single-choice question and two 7-point Likert-scale questions were designed for each category: ‘Have you seen e-cigarette advertisements in this media channel in the past month?’, ‘How much time do you spend on average per day using this media channel in the past month?’, and ‘How much trust do you have in this media channel?’.

### Data analysis

After data collection, the questionnaire results were organized and coded using Epidata 3.1, and the data were analyzed using Python 3.8. In terms of the analysis method, unlike most studies that treat media-related questions separately, this study believes that multiplying the three variables together can effectively measure participants’ exposure to e-cigarette marketing information, both mathematically and logically. Previous health communication studies using the Elaboration Likelihood Model (ELM) have revealed the moderating role of media trust. It indicates that longer media usage does not necessarily mean that individuals are more influenced by the information conveyed through those media. Only when individuals have a high level of trust in the media, the information on particular media will have a more prominent cognitive impact. Specifically, taking print media as an example, if participants have seen e-cigarette marketing information in these media, it is counted as 1. This value is then multiplied by the usage duration and further multiplied by media trust to determine the impact level. If participants have not seen e-cigarette marketing information, the impact level is directly calculated as 0. The specific calculation formula is as follows:

Media influence_i_ = ad exposure_i_ × time of media use_i_ × trust of media_i_

Regarding susceptibility to e-cigarette use, previous studies often employed logistic regression for analysis^21^. However, from a methodological perspective, traditional logistic regression has limited generalizability compared to machine learning models in predictive contexts. Therefore, this study chose the extremely randomized trees model^22^ to predict the susceptibility to e-cigarette use.

For feature selection, this study considered the influence level of e-cigarette marketing information from the five media channels, gender, alcohol consumption, frequency of going to bars, satisfaction with personal life, whether the individual engages in moderate-to-high-intensity exercise weekly, monthly living expenses, parental smoking status, friends’ smoking status, and friends’ e-cigarette use as input features. The binary classification of e-cigarette susceptibility was considered as the output feature. The dataset was split into a 75:25 training set and testing set, and this study used grid search with cross-validation to find the optimal hyperparameters.

Partial dependence analysis is a method that reflects the importance of input features on output labels. This study utilized the feature importance scores of the partial dependence analysis to explore the association between predictors and susceptibility to e-cigarette.

## RESULTS

### Participant characteristics

A total of 303 college students (aged 15–24 years, mean about 19.8 years) were included in the analysis. Table 1 summarizes their demographic characteristics. Briefly, the sample had a higher proportion of females (74.3%) than males (25.7%), reflecting the gender mix of respondents. The vast majority (89.1%) were undergraduate students, with 7.3% pursuing graduate degrees and only 3.6% at junior college level. Virtually all participants (95.4%) were unmarried, as expected in this age group. In terms of personal finances, about 62% reported monthly living expenses in the range of 1501–2500 RMB (1000 Chinese Renminbi about US$140), with roughly 25% spending ≤1500 RMB, and fewer than 13% spending >2500 RMB. This suggests most students had a modest budget typical of college youths in China.

**Table 1:** Sample demographics and background characteristics (N=303)

*Characteristics*	*Category*	*n (%)*
**Gender**	Male	78 (25.7)
	Female	225 (74.3)
**Education level**	Junior college	11 (3.6)
	Undergraduate	270 (89.1)
	Master’s	21 (6.9)
	Doctoral	1 (0.3)
**Marital status**	Married	14 (4.6)
	Unmarried	289 (95.4)
**Monthly living expenses** (RMB)	≤1500	75 (24.8)
	1501–2500	189 (62.4)
	2501–3500	30 (9.9)
	3501–4500	5 (1.7)
	4501–5500	2 (0.7)
	>5500	2 (0.7)
**Alcohol consumption**	Never	1 (0.3)
	≤1 time per month	131 (43.2)
	2–4 times per month	154 (50.8)
	2–3 times per week	15 (5.0)
	≥4 times per week	2 (0.7)
**Bar/nightclub visits** (past 3 months)	Yes	43 (14.2)
	No	260 (85.8)
**Parental smoking**	Yes	180 (59.4)
	No	123 (40.6)
**Friends who smoke**	Yes	165 (54.5)
	No	138 (45.5)
**Friends who use e-cigarettes**	Yes	112 (37.0)
	No	191 (63.0)

RMB: 1000 Chinese Renminbi about US$140.

Notably, almost all students had some history of alcohol use. Only 1 individual (0.3%) ‘never’ drank alcohol, while 43.2% drank ≤1 time per month and about half (50.8%) drank 2–4 times per month. Regarding nightlife, 14.2% of participants reported visiting a bar or nightclub in the past 3 months. Over half of the sample had family or friends who smoke tobacco: 59.4% had at least one parent who is a smoker, and 54.5% had friends who smoke. Fewer reported having friends who use e-cigarettes (37.0%). These contextual factors provide a backdrop for understanding susceptibility. All 303 participants were never users of e-cigarettes by design, but based on their responses to the susceptibility questions, a subset had varying degrees of openness to trying e-cigarettes.

### Predictors of e-cigarette susceptibility

**Figure 1:**
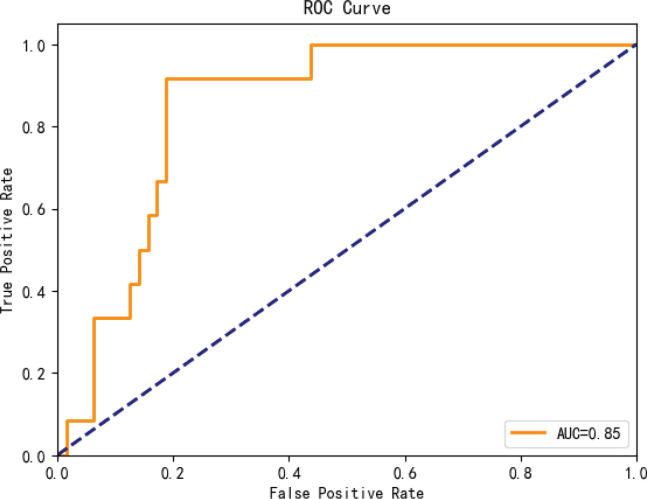
ROC analysis on the test set

The ExtraTrees classifier achieved an accuracy of 81% on the testing set (Figure 1), indicating good model performance in predicting susceptibility status. Compared to logistic regression (accuracy=0.74, AUC=0.44), the Extremely Randomized Trees Model improved the accuracy score by 10%.

**Figure 2:**
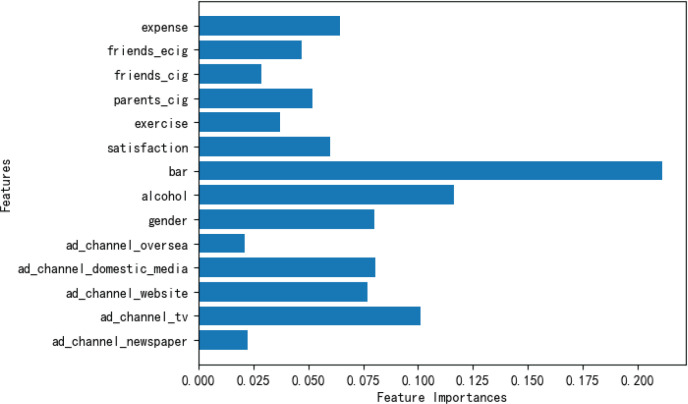
Partial dependence plots for top predictors of e-cigarette susceptibility

Figure 2 presents Influential Factors, and the feature importance results from the model (via Gini importance and partial dependence plots). Several notable predictors are emerged as below.

*Social media exposure*

Among the five media channels, domestic social media exposure to e-cigarette marketing had the strongest relationship with susceptibility. Students who frequently saw e-cigarette content on platforms like WeChat or Weibo and trusted these sources tended to have higher susceptibility scores. By contrast, exposure via foreign social media (e.g. Instagram) and print media showed minimal effect on susceptibility in this sample. This likely reflects the reality that Chinese domestic social networks are a primary conduit for e-cigarette marketing and information in this population. Efforts to regulate e-cigarette promotions on these platforms could thus directly impact youth susceptibility.

*‘Quasi-deviant’ behaviors*

Engaging regularly in alcohol use and bar-going was significantly linked to being in the high-susceptibility group. In a chi-squared analysis, students who had visited bars in the last 3 months were far more likely to be susceptible (χ^2^=7.293, p<0.01). Similarly, those who drank alcohol more frequently, showed higher susceptibility on average. Our interdisciplinary framing considered these excessive behaviors as ‘quasi-deviant’^15^ and indeed they appear to cluster with openness to e-cigarettes. The model’s partial dependence curves indicated that as alcohol use frequency increased (from never up to weekly), the probability of high e-cigarette susceptibility rose accordingly. Those who regularly drank and attend bars constituted a clear high-risk subset. This finding aligns with patterns observed in Western college populations where substance use behaviors co-occur.

*Peer influence*

Having friends who use e-cigarettes was one of the strongest predictors of a student’s susceptibility. The model found that if a student reported any close friends who vape, their likelihood of being susceptible more than doubled, holding other factors constant. In fact, e-cigarette use by friends had a greater impact than having friends who smoke traditional cigarettes or having parents who smoke, which is consistent with the idea that peer modeling of the specific behavior (vaping) is highly influential. Only 7 participants had a parent who vaped, so parental e-cigarette use was not analyzed; however, parental smoking did have a modest effect – students with smoking parents were slightly more inclined toward vaping, perhaps due to a normative acceptance of nicotine use at home, though this was weaker than peer effects.

*Demographics*

Gender and personal spending level showed notable effects. Male students were more likely to be susceptible to vaping than females. This mirrors other studies in China and elsewhere where males tend to have higher experimentation rates with e-cigarettes. Higher monthly living expenses (which could indicate greater disposable income) were also associated with higher susceptibility – possibly because those students can afford e-cigarettes more readily or take part in social activities where vaping might occur. The partial dependence plot for expenses suggested a threshold: students in the top spending tier had a marked jump in predicted susceptibility compared to those with very low monthly funds.

*Other factors*

Somewhat surprisingly, exposure to e-cigarette advertising on websites and traditional radio/TV did not show strong independent effects in the model. It may be that these media channels are less relevant for this demographic (college students likely engage less with TV/radio and more with social media). Personal life satisfaction and exercise habits also did not emerge as significant predictors in the final model, suggesting that general well-being factors were less tied to e-cigarette interest than the specific social and behavioral factors above.

## DISCUSSION

This interdisciplinary study found that exposure to e-cigarette marketing messages, social media use, peer e-cigarette use, parental smoking and ‘quasi-deviant’ behaviors were all significant predictors of susceptibility among Chinese college students. An ensemble machine learning model classified high- versus low-susceptibility students with 81% accuracy, demonstrating the utility of combining these factors to identify at-risk individuals before vaping initiation.

### Exposure to e-cigarette marketing messages and social media use

Exposure to e-cigarette marketing (FI=0.08) and general social media engagement (FI=0.08) were each robust predictors. This aligns with studies showing that youth who view e-cigarette ads on platforms like WeChat or Douyin have higher intent to vape^23^. Despite China’s advertising bans, unregulated promotions persist via influencer partnerships and user-generated content. Strengthening enforcement of digital marketing restrictions and deploying counter-marketing campaigns on social media could reduce youth susceptibility.

### Peer and parental smoking influence

Peer vaping (FI=0.05) significantly raised susceptibility, consistent with social learning theory and empirical US data showing strong peer effects^24^. In our sample, nearly 37% had friends who vape, underscoring the importance of peer-led prevention strategies. Parental smoking (FI=0.05) had a smaller but meaningful effect, suggesting that family norms around nicotine use also shape youth attitudes. Family-based education may therefore complement peer interventions.

### Gender and susceptibility

Although not among the highest feature importances, male gender (FI=0.06) was associated with greater susceptibility, reflecting broader tobacco use patterns in China and risk-taking tendencies among young men. Tailoring messaging to address male-specific motivations – such as stress relief or social identity – may enhance prevention efficacy.

### ‘Quasi-deviant’ behaviors

In our interdisciplinary model, ‘quasi-deviant’ behaviors – specifically, regular alcohol consumption (FI=0.12) and bar attendance (FI=0.21) – emerged as the strongest predictors of e-cigarette susceptibility. Such behaviors deviate from normative student conduct yet are socially tolerated, creating environments where vaping may be more readily accepted. This mirrors findings from US college samples, where nightlife and drinking contexts co-occur with higher vaping rates^25^. In China, bars often host informal vaping promotion and peer-led experimentation. Integrating e-cigarette prevention within broader substance-use programs – such as motivational interviewing that addresses both alcohol use and vaping – could mitigate these interconnected risks.

Our findings are generally consistent with other recent research on e-cigarette risk factors among youth and young adults. A scoping review of Asian adolescents noted that vaping was positively associated with alcohol use and peer tobacco use across multiple studies^13^. Another national survey of Chinese adults aged 18–44 years in 2020–2021, found e-cigarette use was higher among those who were male, had higher income, and those who were current drinkers^9^ – paralleling the demographic and behavior trends we observed for susceptibility. However, our study extends the literature by jointly analyzing these factors through a machine learning lens, which demonstrated the interplay and relative importance of each factor. It also, to our knowledge, is one of the first to apply an ensemble ML model in the context of e-cigarette uptake prediction in a Chinese population. This innovative approach yielded a high predictive accuracy, suggesting such models could be useful in future research for identifying at-risk youth with greater precision than traditional regression models.

### Policy and practice implications

From a policy perspective, the strong influence of social media and peer environments suggests that regulations and public health campaigns need to be multifaceted. Stricter monitoring and penalization of illicit e-cigarette promotions on domestic social media platforms are warranted. Our data imply that current controls are not fully effective, since students still report encountering a lot of content. Schools and universities should also be vigilant; although marketing of tobacco products is banned on campuses, e-cigarette influence can infiltrate through student peer networks and off-campus activities. Education efforts could highlight the manipulative nature of e-cigarette marketing (as youths in one study suggested, more restrictions are needed to protect them)^26^ and correct any misperceptions (e.g. that ‘vaping is harmless’) that marketing might foster. These findings illustrate how environmental exposures (marketing, social settings) and social influences (peers, family) converge with personal behaviors (drinking, bar-going) to elevate susceptibility. Mechanistically, repeated ad exposure may normalize vaping, peer use creates social pressure, and alcohol lowers inhibitions for experimentation. Comprehensive prevention should combine digital advertising enforcement, peer-norm interventions, and substance-use education. Universities can implement routine screening for susceptibility, using brief scales to identify students who may benefit from targeted counseling.

### Limitations

Given the novel application of machine learning in e-cigarette susceptibility prediction, this study employed convenience sampling (n=303) which served as an initial exploration of our interdisciplinary analytical framework. The cross-sectional design precludes causal inferences between predictors and e-cigarette susceptibility, as temporal relationships and potential confounding factors cannot be fully disentangled. While this sampling approach allowed us to validate the extremely randomized trees model’s effectiveness (81% accuracy), the relatively small sample size raises inherent concerns about machine learning applications in limited-data contexts, including potential overfitting risks and instability in feature importance rankings. Future research could employ probability sampling to further strengthen the generalizability of these findings across diverse college student populations.

## CONCLUSIONS

Our research emphasizes that preventing e-cigarette uptake among college students requires interfering with the development of susceptibility before actual use begins. The significant predictors – social media marketing exposure, peer use, male gender, alcohol use and bar-going – point to a mix of environmental and individual levers for intervention. By reducing young people’s exposure to e-cigarette promotions (especially on social media), strengthening peer-led anti-vaping norms, and addressing co-occurring risk behaviors, stakeholders can collectively curb the appeal of e-cigarettes. These findings highlight the importance of comprehensive strategies involving families, schools, and public policy to create a social environment where the healthiest choice (not to vape) is the easiest choice for youth. Continued surveillance of e-cigarette susceptibility and longitudinal research will be important to evaluate the impact of these preventive actions and to adapt them to emerging trends in tobacco product use.

## Data Availability

The data supporting this research are available from the authors on reasonable request.
